# Reestablish immune tolerance in rheumatoid arthritis

**DOI:** 10.3389/fimmu.2022.1012868

**Published:** 2022-09-30

**Authors:** Ziqiang Shuai, Shuang Zheng, Kang Wang, Jian Wang, Patrick S. C. Leung, Bin Xu

**Affiliations:** ^1^ Department of Sports Injury and Arthroscopic Surgery, The First Affiliated Hospital of Anhui Medical University, Hefei, China; ^2^ Department of Rheumatology and Immunology, The First Affiliated Hospital of Anhui Medical University, Hefei, China; ^3^ Department of Neurology, The First Affiliated Hospital of USTC, Division of Life Sciences and Medicine, University of Science and Technology of China, Hefei, China; ^4^ Division of Rheumatology/Allergy and Clinical Immunology, University of California, Davis, Davis, CA, United States

**Keywords:** rheumatoid arthritis, immune tolerance, stem cell transplantation, immune tolerogenic dendritic cells, immune tolerogenic vaccination, treatment

## Abstract

Rheumatoid arthritis (RA) is a chronic progressive autoimmune disease. Despite the wide use of conventional synthetic, targeted and biologic disease modifying anti-rheumatic drugs (DMARDs) to control its radiological progress, nearly all DMARDs are immunologically non-selective and do not address the underlying immunological mechanisms of RA. Patients with RA often need to take various DMARDs long-term or even lifelong and thus, face increased risks of infection, tumor and other adverse reactions. It is logical to modulate the immune disorders and restore immune balance in patients with RA by restoring immune tolerance. Indeed, approaches based on stem cell transplantation, tolerogenic dendritic cells (tolDCs), and antigen-based tolerogenic vaccination are under active investigation, and some have already transformed from wet bench research to clinical investigation during the last decade. Among them, clinical trials on stem cell therapy, especially mesenchymal stem cells (MSCs) transplantation are most investigated and followed by tolDCs in RA patients. On the other hand, despite active laboratory investigations on the use of RA-specific peptide-/protein-based tolerogenic vaccines for T cell, clinical studies on RA patients are much limited. Overall, the preliminary results of these clinical studies are promising and encouraging, demonstrating their safety and effectiveness in the rebalancing of T cell subsets; particular, the recovery of RA-specific Treg with increasing anti-inflammatory cytokines and reduced proinflammatory cytokines. Future studies should focus on the optimization of transplanted stem cells, the preparation of tolDCs, and tolerogenic vaccines with RA-specific protein or peptide, including their dosage, course, and route of administration with well-coordinated multi-center randomized clinical control researches. With the progress of experimental and clinical studies, generating and restoring RA-specific immune tolerance may bring revolutionary changes to the clinical management of RA in the near future.

## Introduction

Rheumatoid arthritis (RA) is an autoimmune chronic disease, primarily characterized by synovial inflammation (synovitis), which further leads to cartilage damage and bone erosion. Extra-articular damages caused by systemic inflammation are very common in RA. If untreated properly, chronic RA can lead to disability and extra-articular multiple systemic damages, some of which may be even life-threatening (1, 2). From the 1890s, when the first nonsteroidal anti-inflammatory drug (NSAID) Aspirin was chemically synthesized and used for RA treatment and glucocorticoids (GCs) was applicated in RA therapy in the 1940s, the goal of RA treatment was directed at symptomatic relief (3, 4). In the 1980s, methotrexate (MTX), the first disease modifying anti-rheumatic drug (DMARD), was approved for RA therapy; however, NSAIDs and GCs could not effectively prevent the radiologic progression of RA (5, 6). With an increasing understanding of RA pathogenesis, the treatment methods for RA and their effects have been much diversified and improved. So far, DMARDs have developed from conventional synthetic DMARDs (csDMARDs) to biological DMARDs (bDMARDs) and targeted synthetic DMARDs (tsDMARDs) with rapid effect and high efficiency (1, 7). Nevertheless, all existing DMARDs cannot overcome the adaptive immune disorders underlying RA. In order to maintain the disease stability and delay disease progress, RA patients have to endure various adverse reactions from DMARDs lifelong while taking a variety of DMARDs (8, 9). Therefore, it is crucial to explore novel treatment aiming to alleviate immune disorders and restore immune balance in RA patients. Here, we will briefly describe present clinical approaches in RA treatment and further discuss the current research progress on reestablishing immune tolerance in RA.

## Conventional therapeutical approaches

**Figure 1:**
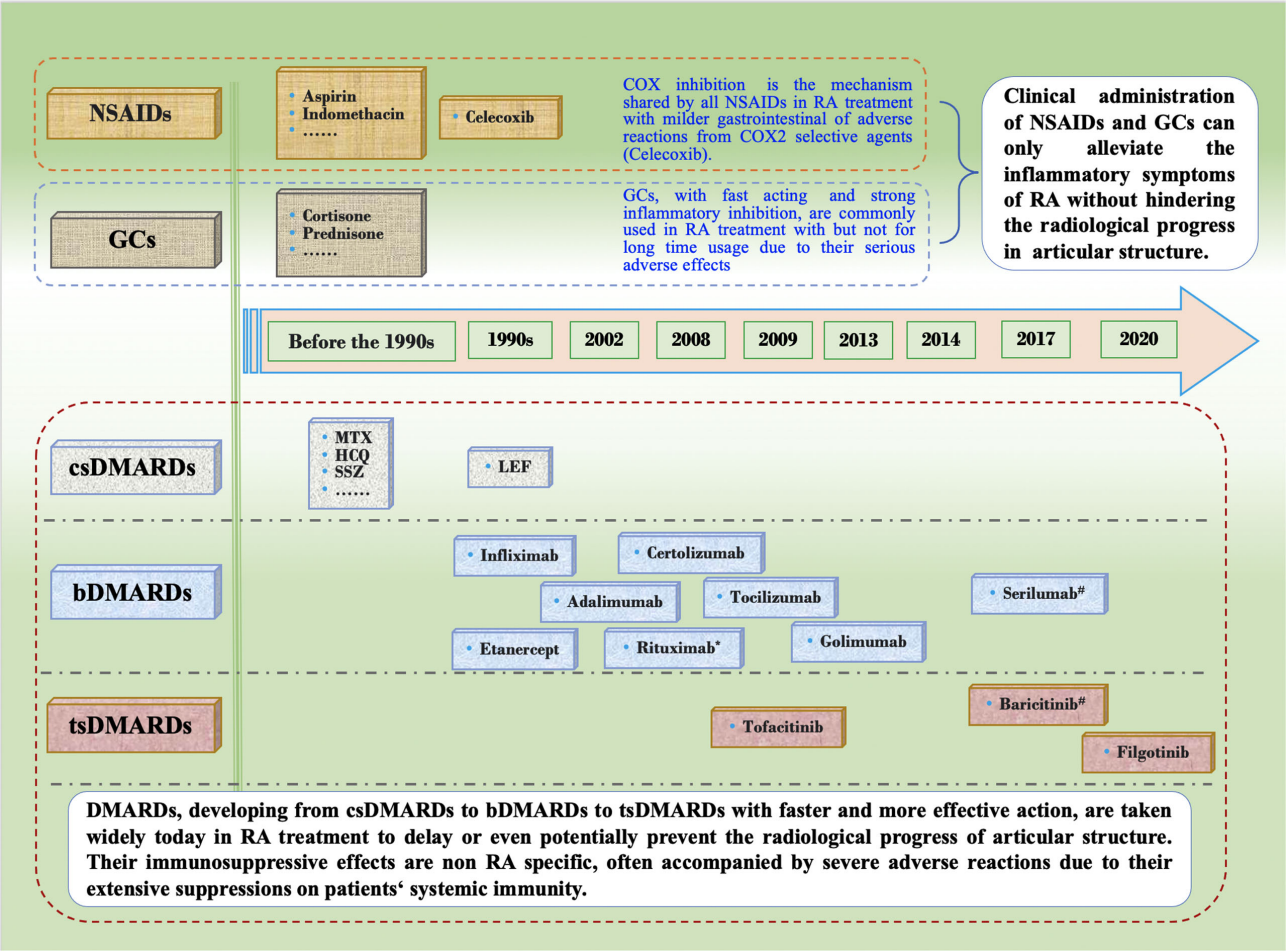
Development of Conventional Agents in the Treatment of Rheumatoid Arthritis. NSAIDs and GCs have stagnated for more than 20 years mainly due to their mere ability to alleviating RA inflammatory symptoms without interfering with the progress of articular erosion. Since DMARDs was proven to be able to slow down the erosive progress, they have been widely used in RA treatment as essential drugs, and have developed from csDMARDs to bDMARDs to tsDMARDs. Choice of csDMARDs, also known as slow acting antirheumatic drugs with non-targeted immunosuppressive effect, have remained the same for the last two deacdes. hbDMARDs, composed of recombinant monoclonal antibodies and receptors of proinflammatory cytokines, were rigorously developed in about the 2000s. tsDMARDs, targeting intracellular various signaling pathways, are becoming widely used in the recent 10 years due to its fast onset, high effect and more convenient oral medication. However, the immunosuppressive effects of all conventional medications above are not RA specific, and therefore cannot correct the autoimmune disorder to cure RA while they have the risk of serious adverse reactions after long-term use. Restoring the disordered autoimmune balance by reconstruction of RA specific immune tolerance is an ideal approach to cure RA. *, approved firstly by Food and Drug Administration (FDA) of USA for refractory RA treatment in 2006. ^#^, approved by European Union but not FDA for RA treatment. NSAIDs, non-steroidal anti-inflammatory drugs; GCs, glucocorticoids; DMARDs, disease modifying anti-rheumatic drugs; csDMARDs, conventional synthetic DMARDs; bDMARDs, biological DMARDs; tsDMARDs, target synthetic DMARDs; COX, cyclooxygenase; RA, rheumatoid arthritis; MTX, methotrexate; HCQ, hydroxychloroquine; SSZ, sulfasalazine; LEF, leflunomide.

Agents treating RA have been increasing and varying, ranging from NSAIDs and GC that mainly alleviate symptoms and inflammation reactions, to DMARDs that can effectively retard and even stop the destruction of joint structure (**Figure 1**). Their characteristic efficacy and various adverse reactions in RA treatment are outlined below.

### Non-steroidal anti-inflammatory drugs and glucocorticoids

NSAIDs are the oldest anti-rheumatic agent that has been used for over 100 years, and are still widely used in the treatment of rheumatism due to their analgesic and anti-inflammatory effects (3). The principal mechanism shared by nearly all NSAIDs is to suppress inflammation in RA by inhibiting the activity of cyclooxygenase (COX) to produce prostaglandins, the common inflammatory mediators. However, it has been verified that all NSAIDs cannot affect disease progression (3). Compared with NSAIDs, GCs are more potent in reducing inflammation and arthritis symptoms by inhibiting the transcription of inflammatory genes, reducing production of cell adhesion molecules, and decreasing the key inflammatory mediators (10). Although it was regarded as a breakthrough in RA treatment when firstly used in the 1940s (4), long-term use of GCs may lead to serious multisystem metabolic side effects and increase the risk of infection (4). Therefore, GCs are often used in combination with DMARDs to relieve RA symptoms under the principle of lower dose and short treatment course (11).

### Disease-modifying anti-rheumatic drugs

Existing DMARDs are mainly composed of synthetic DMARDs (sDMARDs) and biological DMARDs (bDMARDs). sDAMRDs can be divided into csDMARDs and tsDMARDs. All DMARDs are able to delay/modify disease progression and improve the prognosis of RA, just as the abbreviated name DMARDs implies (1, 7). Commonly used representative csDMARDs include methotrexate, leflunomide and hydroxychloroquine. They exert their immunosuppressive and regulatory effects through their respective non-target selective pathways (12, 13). bDMARDs approved for RA include four different modes of action: tumor necrosis factor α (TNF-α) inhibition, inhibitors of co-stimulation, interleukin-6 (IL-6) receptor inhibition, and the depletion of B cells (12, 14–16). While benefiting patients, DMARDs inevitably have toxic and side effects such as hair loss, stomatitis, nausea and hepatotoxic anaphylaxis, thrombocytopenia, ocular toxicity and even autoimmune diseases (9, 17–20). RA patients receiving prolonged DMARDs are susceptible to severe infections and malignancies because of the non-selective immunosuppression of DMARDs (21, 22). In addition, both tsDMARDs and bDMARDs are costly (23). Hence, many RA patients cannot adhere to or even have to terminate DMARDs treatment.

Logically, immunomodulatory therapy to restore tolerance is the ideal way to cure RA. Innovative immunotherapy approaches using stem cells, tolerogenic dendritic cells (tolDCs) and immune tolerant vaccines induced by special peptide have attracted attentions. Results from preliminary studies have been encouraging. Here, they are summarized below.

## Immunotherapy approaches to reestablish immune tolerance in RA

The loss of immune tolerance to autoantigens is the culprit of autoimmune diseases including RA. Regulatory T cells (Tregs) play a pivotal role in maintaining self-tolerance while the imbalance between Treg/Th17 and Th1/Th2 cells influence the development of RA (24, 25). In several pre-clinical models of autoimmune diseases, such as RA, experimental autoimmune encephalomyelitis (EAE), and anti-neutrophil cytoplasmic antibody (ANCA)-associated vasculitis (26), the protective role of antigen- specific Tregs was clearly documented. Thus, it is promising to specifically intervene chronic autoimmune disorders *via* restoring the balance of T cell subgroups by enhancing Tregs function (27). For instance, stem cell transplantation has been suggested to be a potential effective treatment for RA for reconstruction of the immune tolerance (24). In addition, CD4^+^Foxp3^+^Tregs and dendritic cells (DCs), are master regulators and major antigen presenting cells, respectively, and are thus key players in maintaining immune tolerance (28). There are numerous tolerogenic vaccine platforms which have been developed to deliver autoantigens to specific antigen presenting cell subtypes, including protein/peptide, nanoparticle, and DNA/RNA-based vaccines (29). Here, we summarize the recent development in mesenchymal stem cells (MSCs), tolDCs, and protein- or peptide-based tolerogenic vaccination as treatment for RA.

### Stem cell transplantation therapy

Mesenchymal stem cells (MSCs) are pluripotent cells with differentiation potential, which can originate from bone marrow (BM), umbilical cord (UC), adipose tissue (AD), peripheral blood, dental pulp and other tissues. They are capable of self-renewal and can differentiate into chondrocytes, adipocytes, osteoblasts and other cell types. In particular, they have marked immune regulatory effects and are regarded as a powerful tool for the restoration of immune tolerance in controlling autoimmune diseases (30).

#### Mechanism of MSCs treatment

**Figure 2:**
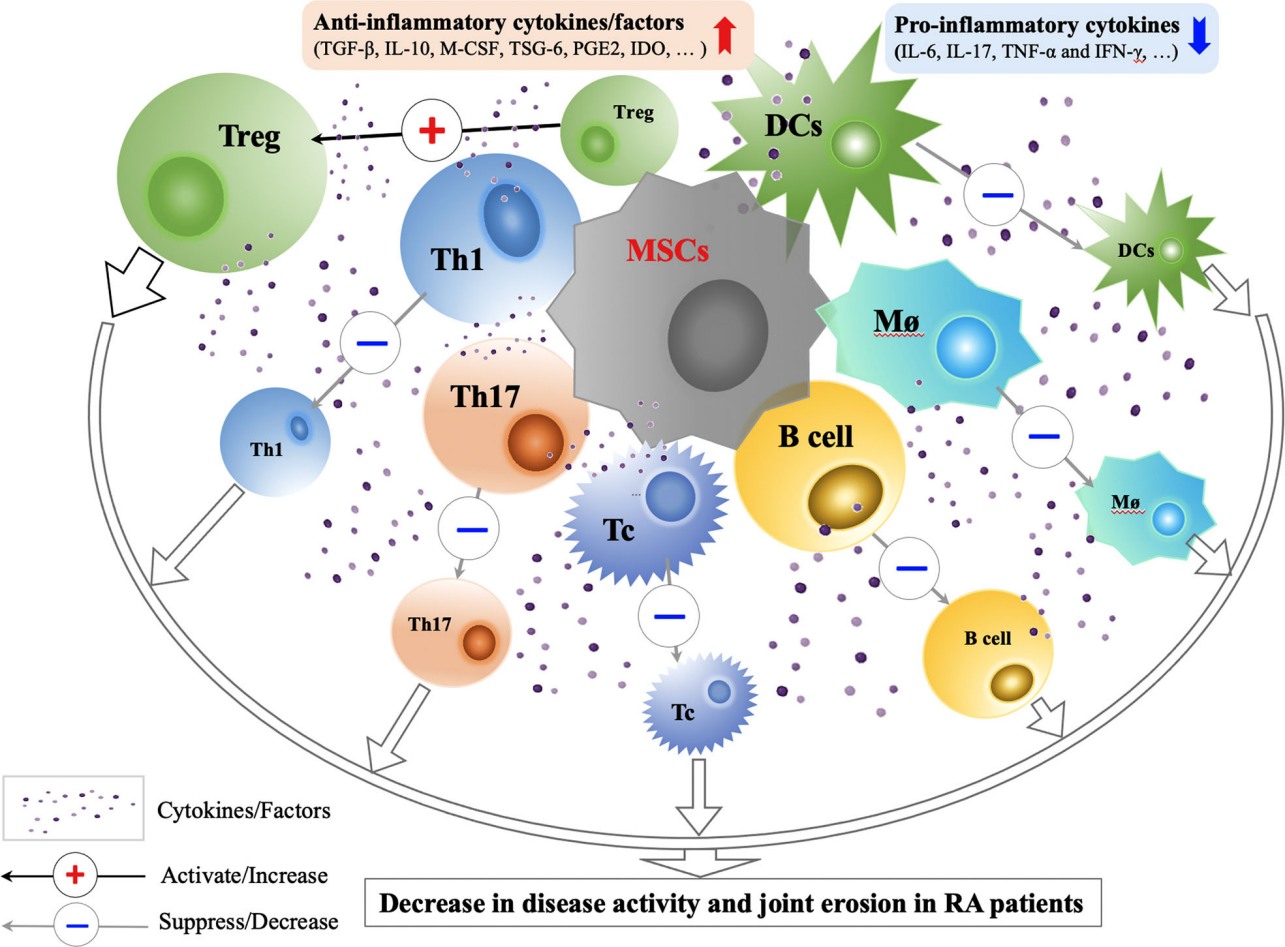
Immunological Mechanisms and Effects of MSCs Transplantation in RA Treatment. Through cell-cell contact and secretion of anti-inflammatory factors (TGF-β, IL-10, M-CSF, TSG-6, PGE2, IDO and etc.), MSCs can improve the functions of Tregs, suppress the activity of Th1, Th17, Tc, B cells, Mø and DCs, correct the immune imbalance between Treg and Th17, and restore the immune tolerance in RA. Animal and preliminary clinical studies on efficacy and safety of MSCs transplantation in RA treatment show that the use of MSCs treatment in RA patients are promising. CCP, cyclic citrullinated; CPR, C-reactive protein; DAS, disease activity score; DCs, dendritic cells; IDO, indoleamine-2,3-dioxygenase; INF, interferon; M-CSF, macrophage colony stimulating factor; MSCs, mesenchymal stem cells; Mø; macrophage; PGE2, prostaglandin E2; Tregs, regulatory T cells; Tc, cytotoxic T cell; TGF, transforming growth factor; TNF, tumor necrosis factor; TSG-6, TNF-stimulated gene-6 protein.

MSCs mainly act through cell-cell contact mediated immune regulation and paracrine mediated immune regulation (31) (**Figure 2**). When MSCs are in the context of proinflammatory cytokines (IL-1β, TNF-α, IFN-γ), their immunosuppressive activity can be enhanced through secretory inhibitory factors, such as TGF-β, prostaglandin (PGE2), indoleamine-2,3-dioxygenase (IDO), or inhibition of effector cells through cell-cell contact and induce the formation of Tregs (32).

Cell to cell contact between MSC and T cells can affect T cell functions in various ways. When MSCs and CD4^+^T cells are co-cultured, BM-MSCs can inhibit the proliferation of CD4^+^T cells and increase the ratio of Foxp3^+^Treg (33). Its ability to promote Treg differentiation depends on IFN-γ level (34). It can also inhibit the secretion of IL-2 and TNF-α by T cells, and restrain its transformation to Th1 cells (33). Using a collagen-induced arthritis (CIA) models in rats, Ma et al. showed that human UC-MSCs (HUCMSCs) therapy could down-regulate RAR-related orphan receptor gamma (RORγt) mRNA and its protein expression, reduce the ratio of Th17 cells, and induce T cell apoptosis. They might up-regulate Foxp3 mRNA and protein expression, and increase Treg cell ratio in the spleen. RORγt and Foxp3 are markers of Th17 and Treg cells, respectively. These findings indicated that the immunomodulation capacity of HUCMSCs could inhibit synovial hyperplasia in CIA rats, with delay in the progression disease, reducing foot swelling and arthritis index (35). Another study found that HUCMSCs had the ability in rats to inhibit the proliferation of spleen T cells and the serum expression of IL-17 and promote serum level of TGF-β (36, 37). Systemic infusion of human AD-MSCs could down-regulate Th1-driven autoimmunity and inflammatory reaction and induce the production of IL-10 in lymph nodes and joints to increase the ratio of CD4^+^ CD25^+^ Foxp3^+^ Tregs (38). A significant increase in the percentage of Tregs was observed one month after MSCs infusion. The increase of Tregs detected by Foxp3 mRNA was parallel to the increase of T-bet and GATA3 transcription factor mRNA levels and the increased levels of IL-10 and TGF-β (39).

BM-MSCs express high levels of toll-like receptor (TLR)-3 and TLR-4, which can determine the phenotypic differentiation of MSCs. Stimulated TLR-4 lead to the production of IL-6, IL-8, and TGF-β and resulted in the development of the pro-inflammatory phenotype of MSC1. Moreover, TLR-3 stimulation of immunosuppressive MSC2 cells can also increase IDO secretion (40). It was found that BM-MSC could inhibit the function and differentiation of B cells and reduce the expression of chemokine receptors during co-culture, including CXCR4, CXCR5, and CCR7 on B cells, thereby inhibiting the chemotaxis of B cells. In the meantime, they could also inhibit the generation of dendritic cells from monocytes (33, 41). Indeed, MSCs therapy resulted in the decrease in levels and proliferation of B cells for at least one year following the MSC treatment in RA patients (42).

With respect to the findings on paracrine effect, the effects of MSCs on Th17/Treg balance have been attributed to various soluble molecules, including IDO, IL-10, PGE2, and nitric oxide (NO) (31, 43, 44). IL-6 and macrophage colony stimulating factor (M-CSF) are produced by MSCs and cause the inhibition of T cell, B lymphocytes, and dendritic cells. This ability of MSCs in releasing anti-inflammatory and anti-apoptotic molecules can effectively protect damaged tissues (38). BM-MSC could inhibit the pro-inflammatory cytokine cells and cytotoxic T cells by modulating anti-inflammatory gene expression, indicating its potential therapeutic effect on molecular level (45). BM-MSC could not only secrete IDO to inhibit T cell proliferation, but could also secret NO synthase to inhibit cell proliferation and to decease the toxicity and secretory function of T cells (33, 41) (**Table 1**). In addition, Ueyama et al. refuted that the levels of proinflammtory cytokines, such as IL-6, IL-17, TNF-α, and IFN-γ, were found to be decreased after the introduction of AD-MSCs in another experimental arthritis animal model. Their study found that HADMSC treatment could inhibit the function of activated inflammatory cells and macrophages, down-regulated inflammatory cytokines, and up-regulated TNF-stimulated gene-6 protein (TSG-6) and TGF-β1 (46).

**Table 1:** Immunomodulatory Effects of MSCs on the T cell subsets.

T cell subsets	Specific regulatory changes	Cytokine levels	Effects of MSCs on T cells	Reference
Th1	T-bet↑	IL-2↓, TNF-α↓, IFN-γ↓	Inhibit T cells transformation to Th1 cells and down-regulate Th1-driven autoimmunity and inflammatory reaction. (The level of T-bet can be influenced by the conventional medications taken at the same time)	(33, 38, 39)
Th2	GATA3↑	IL-4↓, IL-10↑	Not clear	(38, 39)
Th17	RORγt↓	IL-17↓, TNF-α↓	Reduce the ratio of Th17 cells and induce T cells apoptosis.	(35–37)
Treg	Foxp3↑	IL-10↑, TGF-β↑	Promote Treg differentiation and increase the ratio of Tregs by cell to cell contact and by secreting IL-10 and TGF- β in the regulation of autoimmune tolerance.	(33, 38, 39)

IDO, indoleamine-2,3-dioxygenase; MSCs, mesenchymal stem cells; RORγt, RAR-related orphan receptor gamma; Tregs, regulatory T cells.

### Clinical trials of MSCs in RA treatment

In 2010, the first clinical pilot study of MSC in RA therapy was conducted in Korea. Ten patients were enrolled in this study, three of which were RA patient who did not respond to traditional treatment. In this study, isolated and expanded autologous AD-MSCs were used to treat the patients (more than 10^9^ cells after 3 to 4 passages), with one patient receiving intra-articular injection. All patients were followed up for 13 months. The results showed that after treatment, there was significant improvement in the clinical status, and the condition of the patient with intra-articular injection greatly improved, from previously needing crutches for walking to being able to stand up and be off steroids. This study demonstrated that MSC therapy is safe and without severe adverse effect in patients with RA (47).

The first randomized multicenter double-blind placebo-controlled phase Ib/IIa clinical trial of allogeneic AD-MSCs for RA (NCT01663116; **Table 2**) was conducted in Spain in 2011. This study included 53 refractory RA patients with a long history of disease (more than 13 years) and who resistant to at least two biological agents with a DAS28-ESR>3.2. All patients maintained low-dose DMARD, NSAIDs, and/or steroid treatment, but without biologic treatment. The patients were monitored for 6 months. Based on the EULAR criteria, the study had a good response with low DAS28-ESR and C-reactive protein (CRP). According to the authors, the very refractory characteristics of RA patients in the study might have hindered the beneficial effect of MSC treatment. In this study, 19% of RA patients produced MSCs specific anti-human leukocyte antigen I (HLA-I) antibody with no obvious clinical consequences and without anti-HLA-II antibody. This was the first study on the immunogenicity of RA treatment based on allogeneic MSCs (49).

**Table 2:** Summary of Clinical Trials with MSCs in Treating Rheumatoid Arthritis.

Clinical trial identifier	Clinical phase	Status	Source	Registration year	Country	RA patients	MHC context;route of administration	Cells/kg of body weight; number of doses	Estimated/enrolled number of RA patients	Follow-up (Months)	Control group	Ref./estimated completion date
NCT03333681	I	Completed	BM	2016	Iran	Refractory	Autologous; IV	1 to 2 ×10^6^; 1 dose	15	12	No	(48)
NCT01663116	I/II	Completed	AD	2011	Spain	Refractory	Autologous; IV	1, 2 or 4 ×10^6^; 3 doses, weekly	53	6	Yes	(49)
NCT02221258	I	Completed	UC	2014	Korea	Refractory	Allogeneic; IV	2.5, 5 or 10 ×10^7^ 1 dose	9	1	No	(50)
NCT01547091	I/II	Completed	UC	2013	China	Refractory	Allogeneic; IV	4×10^7^/patient; 1 dose	172	36	Yes	(51)
NCT01851070	II	Completed	MPCs	2013	USA	Refractory	Allogeneic; IV	1 or 2 ×10^6^; 1 dose	48	3	Yes	(52)
ChiCTR-ONC-16008770	I	Completed	UC	2016	China	Refractory	Allogeneic; IV	1 ×10^6^; 1 dose	53	12	No	(53)
ChiCTR-INR-17012462	I/II	Completed	UC	2017	China	Refractory	Allogeneic; IV	1 ×10^6^; 1 dose	63	3	No	(54)
NCT03691909	I/II	Completed	AD	2018	USA	Stable treatment	Autologous; IV	Unknown	15	12	No	(55)
NCT01873625	I/II	Unknown	BM	2009	Iran	RA	Autologous; IA	Unknown	30	12	Yes	
NCT01985464	I/II	Unknown	UC	2000	Panama	DMARD- resistant	Autologous; IV	Unknown	20	12	No	June 2020
NCT02643823	I	Unknown	UC	2016	China	RA	Allogeneic; IV	2×10^7^/patient; 4 doses, weekly	40	12	Yes	June 2017
NCT04170426	I/II	Not yet recruiting	AD	2022	USA	Refractory	Autologous; IV	2.0-2.86×10^6^; 3 doses	54	12	No	December 2023
NCT04971980	I/II	Recruiting	UC	2021	China	Refractory	Allogeneic; IV	0.5,1.0,1.5×10^6^; 1 dose	9	28 ± 3 days	No	April 2022
NCT03798028	N/A	Unknown	UC	2017	China	Anemia or pulmonary disease associated	Allogeneic; IV	1×10^6^; 1 dose	250	6	Yes	June 2020
NCT03186417	I	Recruiting	MPCs	2017	USA	During onset	Allogeneic; IV	2, 4, 6×10^6^; 1 dose	20	12	Yes	December 2020
NCT05003934	I	Recruiting	UC	2022	USA	RA	Allogeneic; IV	10×10^7^; 1 dose	20	48	No	September 2025
NCT03828344	I	Recruiting	UC	2020	USA	Refractory	Allogeneic; IV	0.75 or 1.5×10^6^; 1 dose	16	12	Yes	September 2020
NCT02348086	Observational	Unknown	AD	2015	USA	RA	Autologous; IV	Unknown	50	12	No	May 2019
NCT03618784	I/II	Recruiting	UC	2018	Korea	Refractory	Allogeneic; IV	10×10^7^/patient; 3 doses	33	4	Yes	April 2021
NCT04170426	I/IIa	Active, not recruiting	AD	2022	USA	Refractory	Autologous; IV	2.0 or 2.86×10^6^; 1 dose or 3 doses, every 3 days	54	12	Yes	December 2025

AD, adipose tissue; BM, bone marrow; DMARD, disease-modifying antirheumatic drug; IA, intra-articular; IV, Intravenous; MPCs, mesenchymal progenitor cells; MSCs, mesenchymal stem cells; UC, umbilical cord.

There were two large-scale UC-MSCs clinical trials on RA patients in China. One study (NCT01547091, **Table 2**) included 172 refractory patients, 64 of which had a follow-up period of 3 years. All patients maintained low-dose csDMARDs treatment. The study reported a significant remission of disease according to the ACR, DAS-28, ESR and the Health Assessment Questionnaire, with decreased levels of CRP, rheumatoid factor (RF), and anti-cyclic citrullinated peptide (anti-CCP) antibodies, as well as the levels of TNF-alpha and IL-6 in sera. The percentage of Tregs in peripheral blood was increased. This study, for the first time, demonstrated the long-term beneficial effect of MSC-based treatment in combination with low dose of DMARDs for RA patients. No serious adverse effects were reported and only 4% of the patients showed mild adverse effects, such as flu-like symptoms. It would be desirable to conduct a multicenter clinical trial to further validate these enthusing results (51). Another study (ChiCTR-ONC-16008770, **Table 2**) included 53 refractory RA patients. The study was intended to find a marker for determining the effectiveness of MSC treatment. It found that high serum interferon was present in high-reactive RA patients before and 4 weeks after injection, but this change was not seen in non-reactive RA patients. The authors believed that high serum IFN-γ levels are associated with decreased DAS28 values in the responder RA population and claimed that serum IFN-γ levels could be used as biomarkers to predict the clinical benefits of patients (53). These data were consistent with preclinical studies that showed that BM-MSCs might play an immunosuppressive role when they encountered an inflammatory environment in the host. A later clinical trial (ChiCTR-INR-17012462, **Table 2**) also confirmed that BM-MSCs plus interferon greatly improved the clinical efficiency based on MSCs. ACR20 response rates were 53.3% and 93.3% in patients with MSCs monotherapy and with MSCs combined with IFN-γ treatment, respectively. All patients participating in the study were followed up and no unexpected safety problems were observed (54).

Besides the above clinical studies, other clinical trials conducted to date are summarized in **Table 2**. We noted that, firstly, current clinical trials mostly focused on phase I/II studies. The sources of MSCs included BM, AD, and UC (30%, 20% and 50%, respectively) (56). Only a small number of studies adopted allogeneic mesenchymal precursor cells (MPCs) and multipotent progenitor cells in the treatment of refractory RA (MPCs; NCT01851070, **Table 2**) (52). Currently, a clinical trial based on MPCs for the treatment of RA has been launched in the United States (NCT 03186417, **Table 2**). Most trials exhibited good safety results and a few with transient mild symptoms (51, 57, 58). Secondly, the scales of most clinical trials were small. In the early stage, most of the injections were performed intravenously, and only recently, intra-articular injections were conducted. However, improvement could not be significantly sustained beyond 12 months (59). We noted that doses and the use of multiple infusions of bone marrow MSCs seem to be not associated with their beneficial effect, and MSC could tolerate a wide range of doses (56). Thirdly, most of the subjects involved were refractory RA patients while patients in RA early stage and stable treatment period were also included. The general treatment outcome is that the early intervention could produce better effects than later intervention. In addition, there was a study launched to evaluate the improvement of anemia and interstitial pneumonia of RA (NCT03798028, **Table 2**) and the effectiveness for new onset RA (NCT03186417, **Table 2**). Fourthly, not only the clinical effect was evaluated by DAS28, CRP, ESR, RF, anti-CCP antibody, Health Assessment Questionnaire and scores (49, 50, 53, 55), but also patients’ immune balance was evaluated by the ratio of Treg/Th17 cells, serum levels of TNF- α, IL-6, IL-10, and even anti-HLA antibodies (39, 49), which might account for mechanisms of the effect and adverse reactions of MSCs in RA treatment. Lastly, the follow-up times were reported between 1 month to 4 years (**Table 2**). The overall response was that the curative effect was better at 6 months and the introduction of IFN-γ could enhance the effect of MSC. Interestingly, the addition of Chinese herbal medicine was found to improve the anti-inflammatory effect of MSCs (51).

Collectively, based on the current researches, MSCs treatment in RA is safe and promising. However, it still faces the challenges of short-time effectiveness and multiple injections. More multi-center clinical trials are needed to verify the effectiveness of MSCs in RA treatment in the future.

### Tolerogenic dendritic cells therapy

A major objective of DCs-based therapies for RA are to induce and maintain immune tolerance and to restore immune homeostasis by tolDCs (60). To date, tolDCs are mainly derived from monocytes and bone marrow cells, which are obtained by *in vitro* culturing in the presence of GM-CSF, IL-4, by impairing the DC maturation in normal progression and by modulating their specific pro-inflammatory function with various agents loaded (61–63).

The principle behind the treatment with tolDC include directing DCs toward tolerogenic state *ex vivo* and *in vivo*, decrease of effector Th17 cells, reduction of proinflammatory cytokines, and increased number of Treg (64). *In vitro* generation of tolDCs from human monocytes (mo-tolDCs) indicated that tolDCs possess tolerogenic properties (65). Various agents, including dexamethasone, vitamin D3, rapamycin, minocycline, and ethyl pyruvate, were found to contribute to the increased number of human tolDCs *in vitro*, the variation in cytokines secretion, the co-stimulatory protein expressions, as well as the ability to inhibit T cell growth (65). Agents such as dexamethasone, ethyl pyruvate, acetylsalicylic acid, minocycline, and vitamin D3 were also often used to induce tolDCs in mice (66).

#### Animal trials of tolDCs in RA treatment

Advancement in generation of tolDCs were largely developed from animal trials. Collagen induced arthritis (CIA) animal model is a common experimental model for studying the efficacy of tolDCs in RA. It was found that heat-shock proteins (HSPs) loaded tolDC induce increased ratio of type 1 Treg (Treg1) in antigen-specific T cells in RA murine model (67). Additionally, HSP-specific Tregs effectively suppressed established arthritis. HSP peptides could be ideal antigens for tolDCs loading, not only for the treatment of RA, but also for tolDCs-based treatments of other auto-immune diseases. Thus, HSP-loaded tolDCs is worthy of further exploration in RA treatment in the future.

Vasoactive intestinal peptide loaded DCs (VIP-DCs) can contribute to the improvement of arthritis in experimental CIA mouse model (68). VIP-DCs with low expression of MHC-II, CD40, and the costimulatory molecules CD80 and CD86 produce lower levels of pro-inflammatory cytokines. The marked advantages of VIP-DCs on inhibiting inflammation and bone destruction is consistent with a previous clinical study that VIP-DC treatment could clinically inhibit the progression of RA (69).

Subcutaneous administration of microparticle “regulatory vaccine” (REGvac) induced tolDCs could ameliorate the joint arthritis of CIA mice (70). The REGvac contained dendritic cell chemoattractant, potent immunosuppressive molecules and the RA-relevant autoantigen. In REGvac treated mice, IL-6 in the joint tissue was significantly decreased and CD25^+^Foxp3^+^ Treg in the lymph nodes and the spleen increased markedly (70). More interestingly, the IL-10 level was increased while the IL-6 level was decreased. Collectively, this regulatory vaccine targeting tolDCs reversed the progression of joint erosion in CIA mouse and is potentially a promising approach for RA immunotherapy.

Another unique tolDCs approach was based on B cell activating factor (BAFF)-silenced DCs *via* RNA interference techniques (71). BAFF-silenced DCs distinctly suppressed arthritic progression and could re-establish the Th17/Tregs balance in CIA mice. *In vitro*, the regulatory effects of BAFF-silenced DCs on T cells were also verified.

In addition to immature DCs, semimature DCs have also been investigated in RA treatment. Semi-mature DCs could be induced by *Bacteroides vulgatus* and proinflammatory cytokines. These semi-mature DCs would lose their ability to generate proinflammatory cytokines, including TNF-α, IL-1β, and IL-6 (72). In the CIA animal model, DNA-induced-semi-mature DCs were demonstrated to contribute to the increased ratio of Tregs and were effective in preventing CIA (72). It has been reported that both allogeneic tolDCs (allo-tolDCs) and autologous tolDCs (auto-tolDCs) could suppress the expressions of pro-inflammatory molecules and improve the manifestations of arthritis in CIA rats and, therefore, displayed similar efficacy of tolerance between allo- and auto- tolDCs once tolDCs were successfully constructed (73).

#### Clinical trials of tolDCs in RA treatment

The first human phase I study of tolDCs in RA was conducted in 2009 on the safety and efficacy of autologous modified tolDCs exposed to citrullinated peptides (74). This study provided the rationale for advanced research on clinical efficacy of autoantigen immunomodulatory therapy in RA. Autologous tolDCs are often isolated from peripheral blood monocytes of RA patients. DCs were cultured in the presence of NF-κB inhibitor and loaded with citrullinated self-peptides, namely Rheumavax (60). Administration of Rheumavax remarkably improved the ratio of Tregs and decreased the number effector T cells (Teff) and reduced the levels of IL-15, IL-29, chemokine CX3C motif ligand 1 (CX3CL1), and CXCL11 in RA patients (75). The decrease in disease activity score (DAS28) and circulating CRP were also evident in RA patients.

The AuToDeCRA trial is an unblinded, phase I trial, investigating the safety of tolDCs administrated by intra-articular injection (76). Before injecting different doses of tolDCs into the inflamed knee, tolDCs needed to be pulsed with synovial fluids containing self-antigens. This is the first trial to administer tolDCs *via* intra-articular route, in which tolDCs were cultured with dexamethasone/vitamin D3 and loaded with autologous synovial fluid. Dexamethasone/vitamin D3 was used to suppress the activity of NF-κB signaling. These tolDCs expressed a low level of co-stimulation proteins CD40, CD86 and CD83, together with decreased MHC-II expression. Significantly, obvious improvements in symptoms were observed in patients treated with the high dose tolDCs and there was no worsening knee flare, which indicated that intra-articula tolDC therapy was well-tolerated and effective. Furthermore, this study firstly revealed the safety of intra-articular injective tolDCs.

CreaVax-RA are semi-mature autologous DCs, which is heterogeneous nuclear ribonucleoprotein A2/B1, pulsed with recombinant protein-arginine deiminase type-4 (PAD4), citrullinated vimentin antigen, and citrullinated filaggrin. Interestingly, a decrease in numbers of IFN–γ producing T cells and autoantibody levels were observed when CreaVax-RA was administrated (60). This was the first clinical study that suggested that the utilization of semi-mature autologous DCs contributed to the control of autoantibody levels.

In fact, the efficacy of antigen-specific tolDC therapy relies not only on the different tolDCs but also on the different methods of administration in clinical studies. For tolDCs migration towards secondary lymph nodes in humans, intradermal injection of tolDCs should be better than injection by other routes (77). However, tolDCs migration by intradermal injection has not been used widely. Animal and clinical trials with tolDCs to treating RA hitherto are summarized in **Table 3**. Collectively, immunotherapies centered on the administration of tolDCs produce promising results as one of alternatives to immunomodulators for RA treatment, given their ability to specifically suppress auto-immune responses without inducing general immunosuppression.

**Table 3:** Animal and Clinical Trials involving tolDCs in RA treatment.

Origin	Immature/semimature	Target cell generation	Efficacy	Reference
PBMC	Immature	NF-κB inhibitor loaded with citrullinated peptides	Increase in Treg ratio; decrease in Teff and multiple cytokines; decrease in DAS28 and CRP;	(74)
PBMC	Immature	dexamethasone/vitamin D3; loaded with autologous synovial fluid	Improvements in symptoms and well tolerated	(76)
PBMC	Semi-immature	pulsed with PAD4, citrullinated filaggrin, and vimentin antigens	Decrease of numbers of IFNγ producing T cells and decreased autoantibody levels	(60)
PBMC	Immature	HSP	Increased ratio of Tr1	(67)
PBMC	Immature	Vasoactive intestinal peptide	Decreased levels of proinflammatory cytokines; inhibition of inflammation and bone destruction	(68)
PBMC	Immature	Microparticle regulatory vaccine	Increase inTreg cells and IL-10; decrease in IL-6	(70)
PBMC	Immature	BAFF-silenced DCs	Suppression of arthritic progression and re-establishment of the Th17/Treg balance	(71)
PBMC	Semi-immature	Bacteroides (*Bacteroides vulgatus)*	Reduction of proinflammatory cytokines	(72)
PBMC	Semi-immature	DNA	Increase of Tregs ratio and effective in preventing CIA	(72)

BAFF, B cell activating factor; CRP, C-reactive protein; CIA, Collagen-induced arthritis; DAS28, Disease Activity Score; DCs, dendritic cells; HSP, heat-shock proteins; NF-κB, nuclear transcription factor-κB; PAD4, protein-arginine deiminase type-4; PBMC, peripheral blood mononuclear cell; tolDCs, tolerogenic dendritic cells; Tr1, type 1 regulatory T-cells.

### Peptide-based tolerogenic vaccination therapy

Some antigens may have the capability of inducing either an immune response or immunologic tolerance under different exposure conditions and concomitant stimulators (78). Attempts to restore immune homeostasis have been executed with a variety of peptides, especially the disease-related antigenic peptides, and have shown positive effects in various experimental animal models of RA (28). Autoantigens including type II collagen (CII) and proteoglycan (PG) have been proposed as key basements to induce antigen-specific immune tolerance in RA models (78).

A recent research on the therapeutic effect of DerG peptide conjugate vaccines was verified in the human PG G1 domain-induced arthritis (GIA) mouse model of RA (79). The ligand epitope antigen presentation system (LEAPS) was developed by attaching an immune cell binding ligand peptide to a T cell epitope-containing peptide to promote immunogenicity and to determine the resultant response. DerG-PG70 and DerG-PG275Cit vaccines were LEAPS DerG conjugated with the epitope PG70 and the citrullinated form of another epitope (PG275Cit) from PG. Single or combination of subcutaneously administered DerG-PG275Cit and DerG-PG70 vaccines were shown to be protective in the GIA model. Furthermore, splenic T cells and CD4^+^T cells from GIA mice treated with the vaccines preferentially produced anti-inflammatory (IL- 4 and IL-10) rather than pro-inflammatory (IFN-γ or IL-17) cytokine profile in culture. However, the DerG-PG70 (alone or with DerG-PG275Cit) vaccine, but not DerG-PG275Cit vaccine, could induce antibody responses, which indicated that the different peptide vaccines might elicit therapeutic immune responses *via* different immunomodulation. Hence it is critical to select and construct the relevant peptide conjugations as T cell vaccines.

In another study, the long-term protective effect of peptide 90578, a novel fructosylated peptide derived from the immunodominant T cell epitope of bovine CII (bCII) was studied using a bCII and human fibrinogen (FIA-CIA) immunized arthritis mice (80). Intravenous administration of peptide 90578 could lead to significantly beneficial effects on clinical outcome parameters and arthritis histology scores for 12 weeks, as well as improved survival time (80). Additionally, several studies have demonstrated that co-administration of self-antigen with an immunomodulator (e.g., calcitriol, aryl-hydrocarbon receptor ligands, or NF-kB inhibitors) could improve autoimmunity, whereas free antigen could not (26). For example, it has been demonstrated that low dose rapamycin could provide an immunosuppressive microenvironment for tolerance induction by producing anti-inflammatory cytokines and tolDCs. A preclinical study has explored the restoring immune of “tolerogenic polypeptide vaccine” (TPvax), which carried a multiepitope citrullinated peptide and rapamycin in CIA (81). This study demonstrated that TPvax led to increased ratios of secreted anti-inflammatory to pro-inflammatory cytokines and decreased antibody titers.

### Protein-based tolerogenic vaccination therapy

Protein carriers such as monoclonal antibody, cytokines, cells, and pathogen derived immunosuppressive or adhesion proteins have served as both tolerogenic adjuvants and targeting moieties, and have demonstrated efficacy in preclinical models of RA and other autoimmune diseases (82). B29 is a conserved epitope of heat shock protein (HSP) 70, a major ligandome to the MHC-II. It was found that B29-induced CD4^+^CD25^+^Foxp3^+^ T cells could suppress arthritis in the proteoglycan-induced RA mice model both prophylactically and therapeutically, indicating these self-antigen-specific Treg cells could regulate immune disorder *in vivo* (83). HSP 65 is a ubiquitous protein overexpressed in inflamed tissues and capable of inducing immunoregulatory mechanisms. *L. lactis* has probiotic properties and is commonly and safely used in dairy products (84). A recent study has shown that oral co-administration of HSP 65 and *L. lactis* in CIA mouse model ameliorated clinical and histological signs of arthritis, reduced inflammatory cytokines (IFN-γ and IL-17), as well as increased CD4^+^Foxp3^+^Tregs and CD4^+^LAP^+^ T cells (85). This study suggested oral co-administration of HSP 65 and *L. lactis* might be a useful therapeutic approach to regulate inflammatory process of RA.

**Figure 3:**
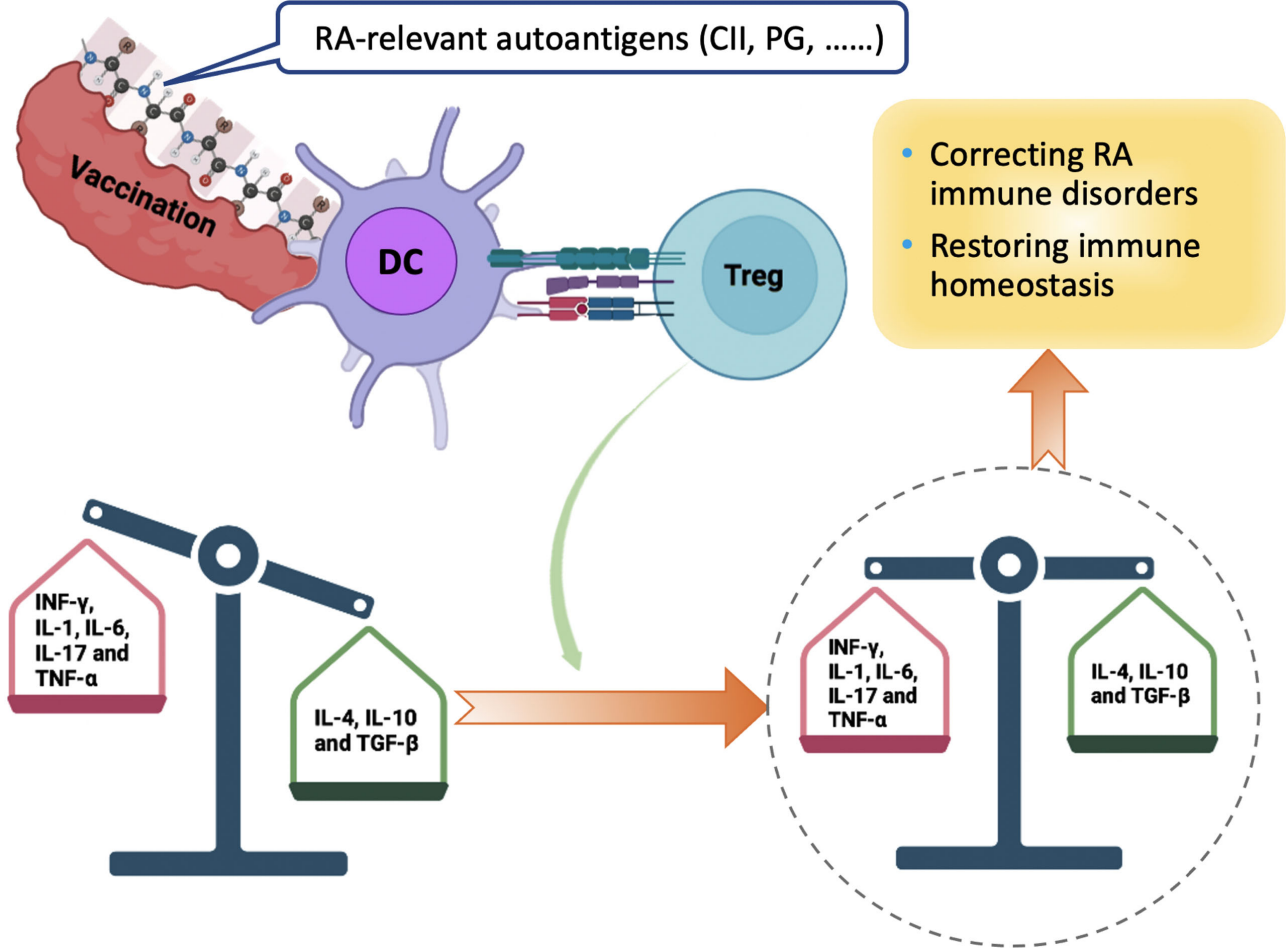
Mechanism of Peptide-based tolerogenic vaccination to Induce the Immune Homeostasis in RA Treatment. Vaccine containing autoantigens related with RA can be processed by DC and presented to Tregs in the context of TCR and cytokines. Activated Tregs function as the main regulator of the immunosuppressive process by increasing the ratio of anti-inflammatory cytokines (green box), restoring immune homeostasis and modulating the balance between pro-inflammatory (red box) and anti-inflammatory cytokines. CII, type II collagen; PG, proteoglycan; DC, dendritic cell; INF, interferon; Tregs, regulatory T cells; TGF, transforming growth factor; TNF, tumor necrosis factor.

There are some recent studies on the mechanism of RA that may be potentially applied to therapeutic vaccines for RA **Figure 3**). The tonicity-responsive enhancer-binding protein (TonEBP) and the 14-3-3ζ protein have been shown the potential therapeutic role in RA models. The TonEBP, which is a Rel family protein involved in the pathogenesis of autoimmune disease and increased inflammation, is required for maturation and function of DCs. This protein is also known as a nuclear factor of activated T-cells 5 (NFAT5). It has been demonstrated that deletion of myeloid cell-specific TonEBP could reduce disease severity in a murine model of CIA and also inhibit maturation of DCs and differentiation of pathogenic Th1 and Th17 cells *in vivo* (86). The 14-3-3ζ protein is an adaptor that can regulate cellular signaling by binding to a wide range of proteins and act as an alarmin. It was recently reported that 14-3-3ζ could cause immune suppression in pristane-induced arthritis (PIA) and CIA models (87). 14-3-3ζ knockout (KO) rats exhibited elevated inflammatory cytokines, particularly for IL-1β and increased bone surface damage. Furthermore, 14-3-3ζ immunization during the pre-symptomatic phase could result in significant suppression of arthritis in both wild-type and 14-3-3ζ KO animals, especially for bone formation. More studies are urgently needed to clarify the underlying molecular mechanisms of inverse vaccination in advancing therapeutic vaccination in RA treatment.

Although previous studies on peptide- or protein- based vaccinations for RA have been encouraging, most of these studies were mainly based on RA animal models, while evidence from clinical studies is still limited. A phase I randomized clinical trial (RCT) was conducted in anti-citrullinated protein Ab (ACPA) positive patients to determine efficacy of three different doses (0.3ml, 1ml and 3 ml) of subcutaneously injected DEN-181, a liposome encapsulating 40mg/ml CII 259-273 peptide and 400ng/ml calcitriol (88). Overall, immunological tolerance was most prominent with the 0.3ml dose, which is also associated with decreased DAS28-CRP and increased naive CII-specific T cells. Further data from this RCT are necessary to determine its efficacy.

## Perspective

Restoring immune tolerance is an ideal but challenging goal in treating autoimmune diseases. In rheumatoid arthritis, experimental studies on the use of stem cell, tolDCs, and T-cell tolerogenic vaccines induced by peptide or protein specific for RA have yielded promising results and are gradually translated to clinical investigations. Current preliminary results showed these therapies are safe and effective. To further develop these therapeutic strategies, it is necessary to focus on how to effectively acquire RA specific stem cells, tolDCs, and T-cell vaccines with increased immunomodulatory capability in down regulating proinflammatory responses. Future work should also focus on the optimization on doses as well as duration and the methods administration, which are validated by large scale clinical trials. In summary, current data from studies on RA treatment by reestablishing immune tolerance are promising and encouraging. We believe that efforts devoted to developing treatment in this direction will transform our current treatment regimen on RA, greatly improve its prognosis and improve the quality of life in RA patients.

## Author contributions

PL originated the topic and supervised the manuscript. ZS wrote the introduction, sections of conventional therapeutic approaches and tolerogenic dendritic cells therapy. SZ wrote the section of peptide-based tolerogenic vaccination therapy and protein-based tolerogenic vaccination therapy. KW wrote the section of stem cell transplantation therapy. JW wrote the perspective. BX wrote the abstract. BX, PL and JW jointly prepared the outline, coordinated the writing of the manuscript and revised the entire manuscript. All authors contributed to the article and approved the submitted version.

## Funding

This study has been supported by the National Natural Science Foundation of China [8180090607] and Anhui Provincial Major Projects of Science and Technology [202103a07020012].

## Conflict of interest

The authors declare that the research was conducted in the absence of any commercial or financial relationships that could be construed as a potential conflict of interest.

## Publisher’s note

All claims expressed in this article are solely those of the authors and do not necessarily represent those of their affiliated organizations, or those of the publisher, the editors and the reviewers. Any product that may be evaluated in this article, or claim that may be made by its manufacturer, is not guaranteed or endorsed by the publisher.

## Glossary

**Table:**

AD	adipose tissue
anti-CCP	anti-cyclic citrullinated peptide
BAFF	B cell activating factor
BM	bone marrow
CCP	cyclic citrullinated peptide
CIA	collagen-induced arthritis
bCII	bovine type II collagen
CII	type II collagen
CRP	C-reactive protein
DAS	disease activity score
DCs	dendritic cells
bDMARDs	biological disease modifying anti-rheumatic drugs
csDMARDs	conventional synthetic disease modifying anti-rheumatic drugs
DMARDs	disease modifying anti-rheumatic drugs
EAE	experimental autoimmune encephalomyelitis
GCs	glucocorticoids
GIA	G1 domain-induced arthritis
HSPs	Heat shock proteins
IDO	indoleamine-2,3-dioxygenase
IFNs	interferons
IL-6	interleukin-6
JAK	Janus-kinase
KO	knockout
LEAPS	ligand epitope antigen presentation system
M-CSF	macrophage colony stimulating factor
MPCs	mesenchymal precursor cells
MSCs	mesenchymal stem cells
NO	nitric oxide
NSAIDs	non-steroidal anti-inflammatory drugs
PG	proteoglycan
PGE2	prostaglandin E2
RA	rheumatoid arthritis
RCT	randomized clinical trial
REGvac	regulatory vaccine
RF	rheumatoid factor
RORγt	RAR-related orphan receptor gamma
STAT	signal transduction and transcriptional activator
tDMARDs	targeted synthetic disease modifying anti-rheumatic drugs
TNF-α	tumor necrosis factor α
tolDCs	tolerogenic dendritic cells
Tregs	regulatory T cells
TSG-6	TNF-stimulated gene-6 protein
TonEBP	tonicity-responsive enhancer-binding protein
TPvax	tolerogenic polypeptide vaccine
UC	umbilical cord
VIP-DCs	vasoactive intestinal peptide loading DCs.
